# New Insights into the Efficacy of Aspalathin and Other Related Phytochemicals in Type 2 Diabetes—A Review

**DOI:** 10.3390/ijms23010356

**Published:** 2021-12-29

**Authors:** Christo J. F. Muller, Elizabeth Joubert, Nireshni Chellan, Yutaka Miura, Kazumi Yagasaki

**Affiliations:** 1 Biomedical Research and Innovation Platform (BRIP), South African Medical Research Council (MRC), Tygerberg 7505, South Africa; christo.muller@mrc.ac.za (C.J.F.M.); nireshni.chellan@mrc.ac.za (N.C.); 2 Centre for Cardiometabolic Research in Africa, Division of Medical Physiology, Faculty of Medicine and Health Sciences, Stellenbosch University, Tygerberg 7505, South Africa; 3 Department of Biochemistry and Microbiology, University of Zululand, KwaDlangezwa 3886, South Africa; 4 Plant Bioactives Group, Post-Harvest & Agro-Processing Technologies, Agricultural Research Council, Infruitec-Nietvoorbij, Stellenbosch 7599, South Africa; joubertL@arc.agric.za; 5 Department of Food Science, Stellenbosch University, Matieland 7602, South Africa; 6 Division of Applied Biological Chemistry, Institute of Agriculture, Tokyo University of Agriculture and Technology, Tokyo 183-8509, Japan; eiyouym@cc.tuat.ac.jp

**Keywords:** aspalathin, bioavailability, diabetes, *C*-glucosyl flavones, gut microbiota, hyperuricemia, inflammation, insulin resistance, mitochondrial dysfunction, oxidative stress

## Abstract

In the pursuit of bioactive phytochemicals as a therapeutic strategy to manage metabolic risk factors for type 2 diabetes (T2D), aspalathin, *C*-glucosyl dihydrochalcone from rooibos (*Aspalathus linearis*), has received much attention, along with its *C*-glucosyl flavone derivatives and phlorizin, the apple *O*-glucosyl dihydrochalcone well-known for its antidiabetic properties. We provided context for dietary exposure by highlighting dietary sources, compound stability during processing, bioavailability and microbial biotransformation. The review covered the role of these compounds in attenuating insulin resistance and enhancing glucose metabolism, alleviating gut dysbiosis and associated oxidative stress and inflammation, and hyperuricemia associated with T2D, focusing largely on the literature of the past 5 years. A key focus of this review was on emerging targets in the management of T2D, as highlighted in the recent literature, including enhancing of the insulin receptor and insulin receptor substrate 1 signaling via protein tyrosine phosphatase inhibition, increasing glycolysis with suppression of gluconeogenesis by sirtuin modulation, and reducing renal glucose reabsorption via sodium-glucose co-transporter 2. We conclude that biotransformation in the gut is most likely responsible for enhancing therapeutic effects observed for the *C*-glycosyl parent compounds, including aspalathin, and that these compounds and their derivatives have the potential to regulate multiple factors associated with the development and progression of T2D.

## 1. Introduction

There are three main types of diabetes, namely, type 1 diabetes, type 2 diabetes (T2D), and diabetes in pregnancy, with T2D accounting for approximately 90% of the total [1,2]. Diabetes, especially global prevalence of T2D, is increasing as reported by the International Diabetes Federation. In 2019, it was estimated that 463 million people have diabetes, and this number is projected to reach 578 million by 2030, and 700 million by 2045 [1]. The rising trend can be attributed to aging, urbanization, and the obesogenic environment [2]. The estimated global direct health expenditure on diabetes in 2019 is USD 760 billion and is expected to grow to a projected USD 825 billion by 2030 and USD 845 billion by 2045 [3].

Natural products from terrestrial and aquatic organisms still constitute huge sources of biologically active factors for the development of drugs, cosmetics and nutraceuticals, as well as functional foods and beverages. Phenolic constituents of plant origin have received extensive attention in recent years, as they are integral to a plant-based diet. Although considered as non-nutritive food components, epidemiological studies suggest that these phytochemicals contribute to general health and well-being [4]. Screening of antidiabetic components from edible natural products and clarifying their modes of actions are considered to be an intelligent policy from the aspects of safety and diabetes prevention, because they have long histories of ingestion every day. This review describes recent studies on preventive and/or alleviating effects of aspalathin, a flavonoid and *C*-glucosyl dihydrochalcone from an endemic South African plant, *Aspalathus linearis*, better known as rooibos, against T2D and diabetes-related disorders. Related flavonoids, some of which are also rooibos constituents, but are also present in other dietary plant sources, are included. Inclusion of the minor rooibos *O*-glucosyl dihydrochalcone, phloridzin [5] is motivated by its structural similarity to aspalathin and potential in managing diabetes [6,7]. Section 2 covers dietary sources, compound stability during food and beverage processing and product storage to contextualize dietary exposure. Furthermore, their bioavailability and microbial biotransformation are addressed, as both are fundamental to the bioefficacy of these compounds. In Section 3, the role of these compounds in alleviating insulin resistance, oxidative stress and inflammation and factors such as gut dysbiosis and hyperuricemia associated with T2D are discussed with the focus largely on the recent literature relating to the bioactivity of the dihydrochalcones and flavones of interest. The antidiabetic, insulin sensitizing, pancreatic β-cell and cardioprotective activities of aspalathin and other major rooibos polyphenolic compounds were extensively reviewed by Muller et al. [8], Johnson et al. [9] and Dludla et al. [10]. The databases that were searched are PubMed, Scopus, Web of Science and Google Scholar with the search limited to the last 5 years, using as search items the following: “aspalathin” or “nothofagin” or “orientin” or “isoorientin” or “vitexin” or “isovitexin” or “phloretin” or “phloridzin” or “phlorizin” and “diabetes” and “inflammation” and “oxidative stress” and “insulin resistance”.

## 2. Aspalathin and Related Compounds—Structures, Sources, Stability, Bioaccessibility and Bioavailability

**Figure 1:**
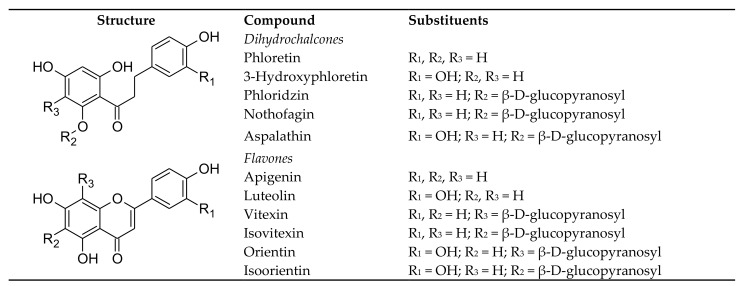
Molecular structures of *C*- and *O*-glucosyl dihydrochalcones and *C*-glucosyl flavones and aglycones.

This section focusses on the rooibos flavonoids, aspalathin, a *C*-glucosyl dihydrochalcone, nothofagin, its 3-deoxy-derivative, their respective *C*-glucosyl flavones, orientin and isoorientin, and vitexin and isovitexin. Contrary to the labile C-O bond of *O*-glucosyl flavonoids, the C-C bond is highly resistant to hydrolysis and degradation. Biotransformation by gut microbiota is necessary to split this bond to release their respective, aglycones luteolin and apigenin [11]. Phloridzin (also known as phlorizin), provides an example of an *O*-glucosyl dihydrochalcone. It shares the same aglycone, phloretin, with nothofagin. The compound structures are depicted in Figure 1.

### 2.1. Dietary Sources

The herbal tea, rooibos produced from *Aspalathus linearis*, is the only dietary source to date of aspalathin. ‘Fermented rooibos’ is the main product on the market, preferred for its flavour. Commercialization of the unfermented (i.e., unoxidized) product, marketed as green rooibos, commenced only after the turn of the 21st century when it was shown to have much higher levels of antioxidants, in particular aspalathin [12]. Consumption of a cup of rooibos (200 mL) would contribute 1.2–36 mg of aspalathin to the diet, depending on the oxidation status of the leaf product, namely fermented or green rooibos (Table 1). Furthermore, natural variation in aspalathin content of the leaves of individual plants (60–135 g/kg dry weight (DW)) [13] manifested in a substantial variation in the aspalathin content of different production batches of green rooibos [14] and, subsequently, extracts (Table 1). The same trends were observed for nothofagin and the flavones. Hot water extracts of fermented and green rooibos for use as food ingredients contain on average 5.8 and 110 g/kg aspalathin, respectively (Table 1). Another study on green rooibos extract showed that the aspalathin content varied between 54 and 116 g/kg DW. The aspalathin content of the extract from the same plant material could be increased to 171 mg/kg when using an 80% ethanol-water mixture [15]. The nothofagin content of green rooibos extract is ca 10 times that of fermented rooibos extract (8 g/kg vs. 0.7 g/kg DW), whereas these extracts had similar orientin and isoorientin levels (≥8 g/kg DW). Vitexin and isovitexin were less abundant than orientin and isoorientin (Table 1).

Nothofagin is not unique to rooibos, but also occurs in other plants, including medicinal plants, such as *Leandra dasytricha* [16] and fruit, namely guava, a tropical fruit, where it occurs together with phloridzin [17]. No quantitative data for nothofagin in guava fruit are available, although it is considered one of the major flavonoids of *Psidium guajava*, commonly referred to as pink guava [17]. Tan et al. [18] reported a phloridzin content of 4.5 and 2.6 mg/kg DW for a white-fleshed and red-fleshed guava varieties, respectively. Phloridzin is present in a variety of plants and fruits, including strawberry, peach, and pomegranate [7]. Apples and apple juice are the main sources of dihydrochalcones in the European diet, estimated at 0.7–7.5 mg phloridzin/day [19]. The Chinese herbal tea, also known as sweet tea (*Lithocarpus polystachus* rehd.), is reported to contain high levels of phloridzin [20].

Apart from rooibos, one or more of the *C*-glucosyl flavones, orientin, isoorientin, vitexin and isovitexin, occur in other dietary sources such as buckwheat [21], mung beans [22], amaranth and quinoa seeds [23], passion fruit [24] and açaí fruit [25]. Vitexin and isovitexin are more abundant than orientin and isoorientin in buckwheat and the compounds are present in much higher levels in the seed hull than the bran and endosperm [26]. A soup from mung beans (*Vigna radiata* L. or previously *Phaseolus radiatus* L.), prepared according to a traditional Chinese cooking method (30 g whole beans cooked for 30 min in 1000 mL water), contains 47.8 mg/L vitexin and 50.7 mg/L isovitexin [22]. The beans are widely used in cuisine. The vitexin and isovitexin content of the bean could vary substantially as demonstrated for commercial samples (0.4–1.5 g/kg and 0.4–1.1 g/kg, respectively) [27]. The seeds of red amaranth (*Amaranthus cruentus* v. Rawa) contain vitexin (0.4 g/kg DW) and isovitexin (0.3 g/kg DW). Orientin and vitexin were present in quinoa seeds (*Chenpodium quinoa*) at 1.1 and 0.7 g/kg DW, respectively [23]. The fruit pulp of yellow passion fruit (*Passiflora edulis* Sims f. *flavicarpa* Degener) contains 16.2 mg/L isoorientin [28], while the pulp of *Passiflora setacea*, a wild species found in Brazil, contains 198, 2116, 55 and 578 mg/kg of orientin, isoorientin, vitexin and isovitexin [29]. White açaí (*Euterpe oleracea*) produces juice with much higher levels of orientin and isoorientin than the purple type [25]. These flavones were the most abundant flavonoids (189 and 90 mg/kg, respectively) in white açaí juice.

### 2.2. Compound Stability during Processing and Product Storage

Processing of plants and fruits creates conditions which affects the stability of polyphenols. Factors such as molecular structure, duration and extent of heat treatment [30], presence of other compounds [31], the chemical potential of the environment and the presence of catalysts such as metals plays a role [32].

Aspalathin is highly susceptible to oxidation, a processing step essential for the development of the characteristic flavour and red-brown colour of ‘fermented’ rooibos [12]. Less than 17% of aspalathin remained in the processed product [33]. Aspalathin is converted to *C*-glycosyl flavanones and flavones (orientin and isoorientin), as well as dimers, benzofurans and tannin-like structures, as previously reviewed [13]. Conversion of nothofagin to vitexin and isovitexin has not yet been studied, but in a study mimicking fermentation of rooibos, the nothofagin content of plant material was reduced at the same rate as aspalathin [34]. Another processing step in the production of fermented rooibos is steam-pasteurization, employed to reduce microbial loads to an acceptable level. The short steam-pasteurization treatment (96 °C/1 min) has little effect on the flavonoid composition of ‘cup-of-tea’ infusions with only a small, but significant reduction in aspalathin content [13].

Interest in aspalathin as a bioactive constituent in rooibos stimulated investigation of its stability during extract production and the heat processing and/or storage of the convenience products, i.e., ready-to-drink (RTD) rooibos iced tea and single-serve and ready-to-use powdered rooibos. Spray-drying of green rooibos extract for production of powdered extract, an ingredient for such convenience products, has little effect on aspalathin degradation as more than 95% was retained in the powder [35]. Factors playing a role in aspalathin stability are the specific formulation of the convenience product, in particular the presence of citric acid and/or ascorbic acid, pH, heating and storage temperature, packaging materials (semi-permeable to moisture and oxygen vs impermeable), and the carrier/ bulking agent [13,36]. Heat processing and storage of RTD rooibos iced tea also reduced the isoorientin content, and to a lesser extent, the orientin content of the beverage with temperature and presence of other food ingredients playing a role [13].

Other studies indicated the relative stability of the *C*-glycosyl flavones and the effect of heat treatment during processing. Pasteurization of passion fruit pulp degrades orientien, isoorientin, vitexin and isovitexin, depending in the severity of the treatment [32]. However, orientien, isoorientin and and isovitexin remained stable during pasteurization (80 °C/60 min) of açaí pulp [37]. Zielinska et al. [21] reported that a hydrothermal treatment of buckwheat which entailed steam treatment and heating (160 °C/30 min) decreased its orientin, isoorientin, vitexin and isovitexin content by 42–47%.

Studies on the stability of phloridzin are limited to apple products. This compound showed high thermal stability in heated apple juice [38,39] and blanched and dried apple pomace [40]. The presence of an extra hydroxy group at C-3 on the B-ring of the dihydrochalcone structure of the 3-hydroxy derivatives of phloridzin decreased their stability compared to phloridzin, while an increase in the degree of glycosylation (from an *O*-monoglucosyl to an *O*-diglycosyl compound) increased stability [38]. Phloridzin was also shown to be more stable than the 3-hydroxyphloretin derivative, 2′-*O*-(6-*β*-D-xylopyranosyl-*β*-D-glucopyranosyl)-3-hydroxyphloretin during storage of dried apple powder [41] and during drying and storage of apple slices [42].

### 2.3. Bioaccessibility and Bioavailability

Phenolic compounds occur in plants either as soluble conjugates or in an insoluble form [43] and they must be released from the plant matrix to be bioaccessibile for intestinal adsorption. They can be released from the plant matrix before ingestion by solvent extraction as in the preparation of tea infusions, by food processing or as part of the digestive process by enzymes, secreted from the intestinal mucosa. Various factors play a role in their bioaccessibility, amongst others the composition of the food matrix and interaction with other food constituents such as macronutrients, as well as gut microbiota. In turn, for the released compounds to be bio-effective, they must be bioavailable and must reach their site of action [44,45].

Poor absorption of flavonoids is predicted by Lipinski’s Rule of Five, the number of rotatable bonds and the polar surface area of the molecule as previously discussed for aspalathin [9], however, as pointed out by Lipinski [46], many natural products, especially natural products from plant origin, do not follow these rules. It is generally accepted that *C*-glycosyl flavonoids such as the rooibos dihydrochalcones and their flavone derivatives are poorly bioavailable compared to *O*-glycosyl flavonoids since the C-C bond, linking the sugar moiety to the aglycone, is resistant to acid hydrolysis and hydrolysis by enzymes that readily cleave *O*-glycosidic linkages. As a result, deglycosylation, considered a critical step in the “activation” of flavonoids following human consumption [47], only occur in the colon due to the action of specific gut microbiota [11]. Despite this caveat, intact *C*-glycosyl flavonoids have been found in the urine of humans following oral ingestion, indicating that their deglycosylation is not a prerequisite for absorption in the small intestine [47]. Microbial biotransformation and catabolism of the flavonoids will be discussed separately from adsorption and liver metabolism.

#### 2.3.1. Aspalathin, Nothofagin and Their Flavones

The in vivo oral bioavailability of aspalathin from rooibos has been the subject of three human studies [48,49,50], following a study on an aspalathin-enriched green rooibos extract, using the pig as model [51]. Vervet monkeys have also been used in a preliminary study on the bioavailability of aspalathin from an aspalathin-enriched green rooibos extract [8] and Bowles et al. [52] investigated the oral bioavailability of pure aspalathin in the mouse. Table 2 was adapted from the review of Johnson et al. [9] and summarizes results for the human studies (Table 2). The human studies showed that aspalathin is predominantly present in the glucuronidated, sulphated and/or methylated form in the urine following phase II liver metabolism after ingestion of a rooibos beverage or aspalathin-enriched green rooibos extract (Table 2). Courts and Williamson [49] showed that 0.74% of the aspalathin from a green rooibos infusion, containing 91 mg per 300 mL, was excreted over a 12 h period in the urine in the form of 3-*O*-methylaspalathin and 3-*O*-methyl aspalathin glucuronide with their concentrations, peaking within 2 h after ingestion of the beverage. Stalmach et al. [50] used the same human subjects to determine aspalathin bioavailability when consumed either in green or fermented ready-to-drink rooibos beverages (500 mL each). It is not clear whether the beverages contained other ingredients normally added to ready-to-drink rooibos such as sugar and citric acid or were in ‘natural’ form. Aspalathin metabolites were excreted in the urine, mainly within 5 h of consumption of the beverages. The presence of non-conjugated aspalathin in the plasma and urine was demonstrated following consumption of a green rooibos infusion delivering 287 mg aspalathin [48]. Subjects also received an isolated fraction of green rooibos, reconstituted in water to a comparable flavonoid content. Blood was sampled from 9 subjects at 0, 1.5 and 3 h after ingestion. Non-conjugated nothofagin was detected in the urine, but not in the plasma. Higher levels of aspalathin were detected in the plasma after ingestion of the rooibos infusion than the isolated fraction, amounting to recovery rates 0.17 and 0.10%, respectively. Some subjects showed no aspalathin in the plasma.

The study of Breiter et al. [48] also provided insight into the absorption of orientin, isoorientin, vitexin and isovitexin after consumption of rooibos. As for aspalathin, the time at which maximum absorption (t_max_) occurred varied between 1.5 and 3 h, depending on the individual and the beverage that was ingested. Their recovery rates varied between 0.18% for isoorientin after consumption of the rooibos tea infusion to 3.45% for vitexin after consumption of the beverage prepared with the isolated fraction. The recovery rates for orientin and vitexin after ingestion of the rooibos beverage was higher than their 6-*C*-glucosyl counterparts, isoorientin and isovitexin, indicating that position of the sugar moiety affects bioavailability. It is worth noting that the content values of the flavonoids in the green rooibos infusion fall within the range obtained for green rooibos infusions prepared from a large number of production batches (Table 1) when compensated for the higher leaf-to-water ratio (10 g/500 mL as opposed to 6.25 g/500 mL) used by Breiter et al. [48].

An interesting finding is that the absorption of the rooibos flavonoids after consumption of the isolated fraction was less than when the same subjects consumed the green rooibos infusion [48]. This was despite comparable intake of total flavonoids, suggesting that the composition of the beverage played a role in the absorption of the flavonoids, an aspect worthy of further investigation, especially in view of a relatively new practice in Japan to brew rooibos using cold water instead of hot water [53].

Transport of aspalathin across the intestinal epithelial layer was postulated to be likely via diffusion [47]. Bowles et al. [52], also using the Caco-2 monolayer cell model, investigated the role of active glucose transporters such as sodium-glucose co-transporter-1 (SGLT1) and glucose transporter 2 (GLUT2), by performing experiments in the presence and absence of the SGLT1, GLUT2 and efflux (P-glycoprotein; P-gp) inhibitors, namely phloridzin, phloretin and verapamil, respectively. These transporters exhibited no effect on aspalathin and its transepithelial flux occurred without evidence of the formation of metabolites [47,52].

In vivo studies on extracts of other plants provided additional insight into the bioavailability of these *C*-glucosyl flavones. A number of studies were performed on the pharmacokinetics of one or more of these compounds. They were mainly detected in the feces of rats, following oral gavage of a single dose of a 10% aqueous solution of bamboo extract (1 g/kg body weight (BW)) [54]. The compounds were excreted after 24 h with cumulative excretions as the parent amounting to 24.04, 24.05, 9.72 and 20.97%, respectively, of the dose for orientin, isoorientin, vitexin and isovitexin. None was detected in the blood and tissues (brain, liver, kidney and thigh muscle). Metabolites indicating microbial degradation in the colon were present in the feces. Intravenous administration of pure orientin to rabbits produced a substantially higher area under the curve (AUC_0–t_) than when present as constituent of *Trollius chinensis* Bunge extract at the same orientin dosage level [55]. This was attributed to the effect of other constituents in the extract, in particular *O*-glycosides of orientin, which were postulated to be transformed to orientin in vivo. Intravenous injection of orientin to rats at 20 mg/kg BW showed its rapid distribution (within 5 min) to various tissues, including the kidney, liver and lungs, followed by a decline. Orientin was eliminated from the plasma within 90 min [56]. Vitexin cleared more rapidly from the blood than orientin when *Trolius ledebourii* extract was administered intravenously to rats [57]. A metabolomics study, using an extract of *Tetrastigma hemsleyanum* leaves administered to rats through oral gavage at a high dose (5 g/kg BW), showed that (iso)vitexin underwent glucuronidation, hydroxylation and methylation. These metabolites were detected in both the plasma and urine [58]. Methyl-(iso)orientin and (iso)orientin-sulphate were detected in the urine, but not the plasma. Several conjugates of the aglycones were detected in the plasma and urine, but their presence could be the result of the aglyones being constituents of the extract.

Tremmel et al. [59] performed in vitro metabolism of the pure *C*-glucosyl flavonoids, using the Caco-2 monolayer model, to gain insight into their metabolites. Vitexin showed limited metabolism and only phase I hydroxylated/methoxylated metabolites were observed. However, orientin, isoorientin and isovitexin were extensive metabolised, with formation of both phase I and phase II metabolites. Cleavage of the C-C glucosidic bond of the latter compounds also occurred, as metabolites of their corresponding aglyones, luteolin and apigenin were detected. Most of the detected metabolites were detected in the apical compartment, suggesting the role of efflux transporters.

#### 2.3.2. Phloridzin and Phloretin

Phloridzin is an *O*-glucosyl dihydrochalone of phloretin (Figure 1), which makes it a substrate for deglycosylation in the small intestine by the epithelial cell β-glucosidase, lactase-phloridzin hydrolase (LPH), releasing phloretin and glucose [60]. Phloretin is rapidly absorbed in the small intestine as shown in rats, reaching maximum plasma concentration (C_max_; 952 ng/mL) after 15 min, when administering 100 mg/kg BW. It had an elimination half-life (t_½_) of 242 min. Phloretin was detected in the heart, lung, liver and kidney, but not the brain [61]. In another study on rats, phloretin appeared in the plasma 10 min after oral administration (100 mg/kg BW), taking at least 5.49 h to eliminate 63.2%. Its oral bioavailability was 8.68% [62]. Crespy et al. [63] reported that when phloridzin is fed to rats in a supplemented diet, it took longer for phloretin to be detected in the plasma than when the diet contained phloretin, but after 10 h, the phloretin concentration was the same, irrespective of the compound ingested. Phloridzin was not detected in the plasma and 85–95% of the circulating forms were glucuronidated and/or sulphated phloretin with the remainder the unconjugated aglycone. Ingestion of either the glucoside or the aglycone resulted in the excretion of 10.4% of the dose in the urine. Oral administration of phloridzin to normal and type 2 diabetic rats demonstrated that the health status of the animals affected its pharmacokinetics [64]. The bioavailability of phloridzin was ca. 5% in the diabetic rats, while it was almost 0% in the normal rats, with only traces detected in the blood. Phase II metabolites were the main forms in the blood of both the normal and diabetic rats. The other pharmacokinetic parameters were also affected, with AUC_0-t_ and C_max_ increased and t_½_ decreased for diabetic rats. Wang et al. [64] postulated that changes in the intestinal tract permeability may be due to up-regulation of lipopolysaccharides (LPS) and down-regulation of P-gp. Zhao et al. In addition, [62] showed that phloretin is a substrate of P-gp and multidrug resistance protein 2 (MRP2).

The plasma concentration of phloretin in human subjects reached a maximum after 3 h, following consumption of apples [65]. Human consumption of a polyphenol-rich juice beverage, containing polyphenols from different sources, including apple, showed the presence of phloretin-2′-*O*-glucuronide in the plasma and urine, while another phloretin-*O*-glucuronide and three phloretin-*O*-glucuronide-*O*-sulphates were detected in the urine [66].

#### 2.3.3. Microbial Biotransformation and Catabolism

**Figure 2:**
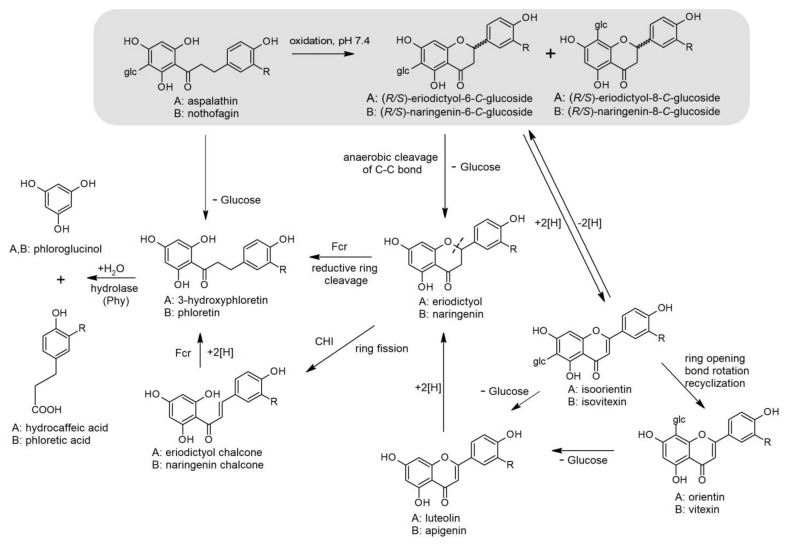
Proposed microbial degradation of aspalathin, nothofagin and their flavones, following non-microbial oxidative conversion at high pH conditions, found in the small intestine [69] (shaded area). The figure was adapted from Muller et al. [8]. Glc = glucose; R = OH for aspalathin and metabolites; R = H for nothofagin and metabolites.

A complex symbiosis exists between polyphenols and gut microbiota. Polyphenols promote the growth of beneficial gut bacteria, while gut microbiota play an essential role in the bioavailability of unabsorbed polyphenols [67]. The resistance of the C-C bond of rooibos *C*-glucosyl dihydrochalcones and flavones to hydrolysis by LPH in the small intestine requires liberation of their aglycones by colonic microbiota. In a previous review paper by our group [8], we proposed a metabolic degradation pathway for aspalathin based on evidence available in the literature, in particular by Braune and co-workers. New information has since come to light, such as the oxygen-sensitive NADH-dependent reductase, flavanone- and flavanonol-cleaving reductase (Fcr) (previously described as enoate reductase), isolated from *Eubacterium ramulus*, that is able to cleave the flavanones, naringenin and eriodictyol, for the formation of phloretin and 3-hydroxyphloretin, respectively [68]. Their presence in the colon may be from the flavone precursors, vitexin/isovitexin and orientin/isoorientin. Another source of eriodictyol could be the flavanone oxidation products of aspalathin, (*R/S*)-6-β-D-glucopyranosyleriodictyol (dihydro-isoorientin), and (*R/S*)-8-β-D-glucopyranosyleriodictyol (dihydro-orientin), formed under oxidative conditions in solution, especially at slightly acid to neutral pH, found in the small intestine [69]. An updated and revised scheme that includes nothofagin is proposed in Figure 2. The conversion of nothofagin to flavanones and flavones is supposition, based on that of aspalathin, discussed in Section 2.2. To date, no evidence of the conversion of nothofagin to its naringenin derivatives and their conversion to vitexin/isovitexin exist. Furthermore, evidence of the conversion of phloretin to naringenin under mild oxidative conditions is also lacking.

Several human colonic bacteria are able hydrolyze the C-C bond of a variety *C*-glycosyl compounds (reviewed by Braune and Blaut [11] and Wei et al. [70]). Bacteria identified to deglycosylate the rooibos *C*-glucosyl flavones are *Eubacterium cellulosolvens* and the bacterial strain CG19-1. *Eubacterium cellulosolvens* removes the glucose in the C-8 position (isoorientin and isovitexin), but not in the C-6 position (orientin and vitexin) [71]. The bacterial strain CG19-1 was, however, able to remove the C-6 sugar moiety [72]. The rate of biotransformation would depend on the specific molecular structure of the *C*-glucosyl flavone as demonstrated by Zheng et al. [73] for orientin, vitexin and isovitexin. *Enterococcus faecalis* W12-1A transformed isovitexin to apigenin within 6 h, but deglycosylation of isovitexin and orientin took much longer (14 and 16 h, respectively). Recent analysis of the Unified Human Gastrointestinal Protein catalogue for genes encoding putative flavonoid-modifying enzymes [74], identified gut bacteria not previously considered to modify flavonoids such as *Agathobacter faecis*. Genes from these bacteria encode enzymes involved in *C*-deglycosylation.

Other bacterial conversions of the flavones include reduction to flavanones (naringenin and eriodictyol), followed by degradation to corresponding chalcones by C-ring fission by chalcone isomerase. The chalcones are reduced to the dihydrochalcones, phloretin and 3-hydroxyphloretin by Fcr. The hydrolysis of the dihydrochalcones by phloretin hydrolase produces amongst others, 3-(4-hydroxyphenyl)-propionic acid (phloretic acid) and 3-(3,4-dihydroxyphenyl)-propionic acid (hydrocaffeic acid), respectively, in addition to phloroglucinol, which is further degraded to the short-chain fatty acids (SCFA), acetate and butyrate. *Eubacterium ramulus* produces the enzymes chalcone isomerase, Fcr and phloretin hydrolase [75,76,77]. It also rapidly degrades phloroglucinol to butyrate and/or acetate [78]. Direct reductive cleavage of the flavanones by Fcr (isolated from *E. ramulus*) to form dihydrochalcones, instead of via a chalcone as intermediate, can also occur (Figure 2). This reaction by NADH-dependent reductase was stereospecific, showing preference for the (*R*)-enantiomer of naringenin and eriodictyol [68]. Bacteria involved are *E. ramulus* and *Flavonifractor plautii* (originally assigned as *Clostridium orbiscindens* [79])*,* shown to catalyze the degradation of luteolin and eriodictyol to hydrocaffeic acid and phloroglucinol [80,81]. A flavone reductase was isolated from *Flavonifractor plautii* (ATCC 49531), which specifically catalyzes the hydrogenation of the C2-C3 double bond of flavones [79]. It is thus clear that irrespective of the parent compound ingested, ultimately the same microbial degradation products, namely phloretic acid/hydrocaffeic acid and organic acids, will form in the colon.

## 3. The Therapeutic Potential of Aspalathin and Related Compounds in Targets for Diabetes from Recent Literature

Phytochemicals, specifically the dihydrochalcones and related compounds, have been shown to target several pivotal pathways associated with glucose and energy metabolism in metabolic disease and diabetes. Recent research into these pathways include the discovery of several key target molecules associated with insulin resistance, muscle metabolism, insulin signaling and sensitivity, glucose reabsorption, gut dysbiosis and pancreatic β-cell protection. These mechanisms are discussed in this section.

### 3.1. Insulin Resistance and Hyperuricemia

The risk factors for T2D include a genetic predisposition, metabolic permutations such as hyperglycemia, dyslipidemia and hyperinsulinemia and environmental factors such as diet and exercise. The main causal factors that fuel T2D development include insulin resistance (IR), inflammation and oxidative stress [82]. Insulin resistance is a metabolic aberration causing an impairment of insulin to achieve its physiological effects, including the stimulation of glucose uptake and inhibition of hepatic glucose output. Recently, IR has also been strongly associated with mitochondrial dysfunction related to defective peroxisome proliferator-activated receptor γ co-activator 1α (PGC 1α) signaling, a key regulator of oxidative metabolism genes [83]. In T2D, disruption of PGC 1α signaling disrupts substrate oxidation [84], resulting in the accumulation of lipotoxic lipid metabolite intermediates such as ceramides and diacylglycerol (DAG) that differentially affects tissues such as muscle, fat, kidney and liver via pro-inflammatory processes and the generation of reactive oxygen species (ROS) [83]. In addition, to compensate for IR, the pancreatic β-cells produce more insulin, but this is at the expense of the negative physiologic effects of hyperinsulinemia such as promoting hepatic steatosis, atherogenesis profile, and, in the kidney, increased sodium retention and reduced renal excretion of uric acid by the proximal tubules, leading to increased blood pressure and hyperuricemia, respectively [85,86,87]. Aspalathin has been shown to ameliorate insulin resistance by enhancing insulin signaling via protein kinase B (AKT) and 5′-Adenosine monophosphate-activated protein kinase (AMPK) activation and by reducing inflammation via protein kinase C (PKC) and nuclear factor-κB (NFκB) pathways. These effects have been demonstrated in both in vitro and in vivo models and has been extensively reviewed by Muller et al. and Johnson et al. [8,9].

**Figure 3:**
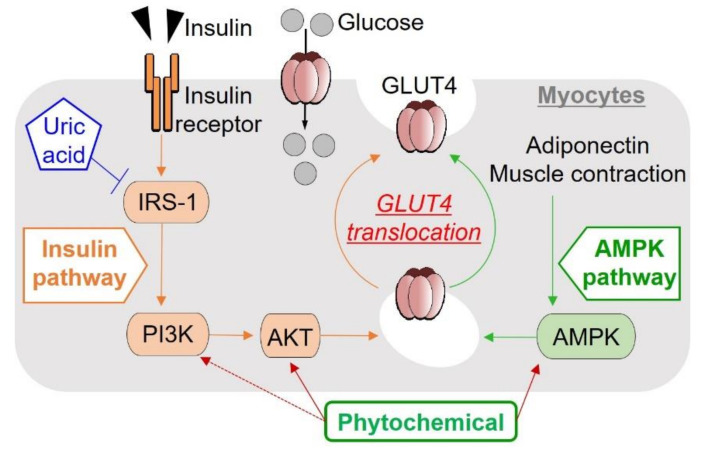
Schematic representation of basic insulin signaling pathways and effects of phytochemicals and uric acid. Phytochemicals, including phenolic compounds described in this review, modulate cellular energy usage by enhancing insulin activity via AKT activiation and activation of the metabolic regulator, AMPK, cumulating in the translocation of GLUT4 to the plasma membrane and increasing glucose uptake and utilization by insulin responsive tissues such as skeletal muscle cells (modified from [93]).

Hyperuricemia is the high uric acid state in blood/plasma /serum and is known as the cause of gout [88]. High uric acid levels directly inhibit insulin signaling (Figure 3) by insulin receptor modification and induces IR [89]. Hyperuricemia also induces endothelial insulin resistance [90]. Aspalathin has been already reported to suppress hyperuricemia by inhibiting xanthine oxidase activity in vitro and inosine-5′-monophosphate (IMP)-induced hyperuricemia in vivo [91]. T2D model KK-A^y^/Ta mice have been found to show hyperuricemia as well as hyperglycemia [92], and taxifolin was demonstrated to suppress these spontaneous hyperuricemia and hyperglycemia, and hence an index of IR such as homeostasis model assessment of insulin resistance (HOMA-IR) [93], as well as purine bodies-induced hyperuricemia [94]. Recently, human serum uric acid concentration was shown to be associated with IR in adults as risk for T2D [95]. Thus, it is of interest to know whether or not aspalathin has the potential to suppress the spontaneous hyperuricemia in T2D model KK-A^y^/Ta mice as an animal model of preclinical trial [92]. The T2D model KK-A^y^/Ta mice seem to be useful for clarifying modes of actions of aspalathin on hyperuricemia, as well as those for the onset of spontaneous hyperuricemia in the whole-body levels.

Quercetin and isorhamnetin have been reported to promote glucose uptake in myocytes [96,97]. Quercetin could lower the plasma uric acid concentration in pre-hyperuricemic human males [98]. Quercetin and isorhamnetin [99] also suppressed purine bodies-induced hyperuricemia in mice. Likewise, urolithin A, a gut microbial metabolite of ellagic acid with anti-inflammatory effect [100], suppressed the increase in plasma uric acid concentration in purine bodies-treated mice [101]. Thus, these phytochemicals and metabolites have a possibility to alleviate IR through hypouricemic actions in the T2D state. Quercetin, apigenin and luteolin as aglycons significantly and dose-dependently suppressed uric acid production in cultured AML16 hepatocytes in vitro. However, vitexin and orientin failed, but rutin (quercetin-3-*O*-rutinoside) succeeded in suppressing both uric acid production in cultured hepatocytes and hyperuricemia induced by purine bodies administration in mice [102]. These differences may be due to cleavage of the C-O-C bond in the buffer or the C-C bond in the intestine of mice as discussed in Section 2. Thus, enzyme(s) produced by bacteria able to cleave the C-C bond may be useful, at least, in the screening test for inhibitors of uric acid production in vitro. In addition to IR [89], hyperuricemia is associated with metabolic syndrome, cardiovascular diseases and chronic kidney diseases [103], as well as β-cell dysfunction [104]. Thus, these studies on the effect of aspalathin on hyperuricemia in the T2D state are of value and interest to metabolic diseases.

### 3.2. Muscle Metabolism

In glucose metabolism, the skeletal muscles account for the majority (~75%) of insulin-mediated glucose uptake in the post prandial state, and hence plays an important role in maintaining glucose homeostasis [105,106]. Skeletal muscle cells and tissues are also involved in metabolism of nutrients such as amino acids and lipids [105] and are, therefore, important for studying effects and mechanisms of various biofactors, including aspalathin and other phytochemicals. Aspalathin was the first phytochemical found to increase glucose uptake in a rapid glucose uptake assay system, which was contrived without the use of radioisotope-labeled glucose in L6 myotubes [107]. In a follow-up screening, aspalathin was found to suppress the increase in fasting blood glucose levels and improve the intraperitoneal glucose tolerance test (IPGTT) of 2TD model *db/db* mice [107]. In further experiments, aspalathin dose-dependently increased glucose uptake in cultured L6 myotubes at concentrations of 0–100 µM. This aspalathin-induced increase in glucose uptake was completely suppressed by 10 µM of Compound C, an inhibitor of AMPK. Thereafter, aspalathin was found to activate (phosphorylate) AMPK, while it did not activate AKT, another promotor of GLUT4 translocation, indicating that aspalathin promoted the translocation of GLUT4 to the plasma membrane of L6 myotubes via the AMPK pathway. In *ob/ob* mice, aspalathin significantly suppressed the increase in fasting blood glucose levels and improved glucose intolerance, and decreased expression of hepatic genes related to gluconeogenesis and lipogenesis, these leading to antihyperglycemic and antihyperlipidemic effects [108]. Aspalathin also reduced the levels of liver triglycerides, serum thiobarbituric acid-reactive substances and tumor necrosis factor-α (TNF-α) in T2D *ob/ob* mice. Although the adiponectin levels were decreased in *ob/ob* mice compared to normal mice, aspalathin prevented this reduction [108,109]. Together these findings strongly suggest that aspalathin has potential antidiabetic value.

A green rooibos extract also increased glucose uptake in cultured L6 myotubes and induced phosphorylation of AMPK [110], similar to aspalathin. In addition to AMPK, the extract also promoted phosphorylation of AKT in L6 myotubes unlike aspalathin, suggesting involvement of extract constituent(s) other than aspalathin in AKT phosphorylation (Figure 3). Promotion of GLUT4 translocation to the plasma membrane in L6 myotubes by the rooibos extract was demonstrated by Western blotting analysis. Subchronic feeding of T2D model KK-A^y^/Ta mice with the same extract suppressed the increase in their fasting blood glucose levels. These results strongly suggest that green rooibos extract has antidiabetic potential through multiple modes of action [110]. Recently, the flavanonol, taxifolin (aslo known as dihydroquercetin), has been reported to activate both AMPK and AKT, to promote GLUT4 translocation to the plasma membrane, and to increase glucose uptake in L6 myotubes, leading to reduction of fasting blood glucose levels in KK-A^y^/Ta mice [93].

### 3.3. Molecular Therapeutic Targets for Glucose Homeostasis, Insulin Signaling and Pancreatic β-Cell Protection

Identifying key regulatory metabolic proteins relevant to glucose and energy metabolism has become the focus for new therapeutics that alleviate metabolic dysfunction. In this section, rooibos-related compounds that inhibit or enhance the activity of key modulators of cellular metabolic processes are discussed as potential therapeutics.

Mammalian sirtuins, and most specifically sirtuin 6 (SIRT6) are essential regulators of glucose homeostasis and insulin sensitivity in peripheral tissues [111]. Suppression of SIRT6 improves insulin activation of AKT and increases glycolysis, whilst suppressing gluconeogenesis by non-repressed protein 5 (GCN5) and PGC-1α activation [112]. However, SIRT6 regulates glucose sensing in pancreatic β cells, and by suppressing SIRT6, in pancreatic β-cells, forkhead box protein O1 (FOXO1) is deacetylated and pancreatic and duodenal homeobox 1 (PDX1) and GLUT2 expression is diminished inducing systemic glucose intolerance [113]. Interestingly, quercetin and luteolin regulated SIRT6 activity by inhibiting its activity at low concentrations (IC_50_ values of 24 and 2 μM, respectively) whilst stimulating SIRT6 activity at high concentrations (EC_50_ values of 990 and 270 μM, respectively) [114]. Rutin, a glycoside derivative of quercetin, showed 60% inhibition SIRT6 in a deacetylation assay at a concentration of 200 μM [115]. Further SIRT6 inhibition by quercetin derivatives, diquercetin and 2-chloro-1,4-naphtoquinone-quercetin (IC_50_ values of 130 μM and 55 μM, respectively), differed by their binding and substrate interactions. However, further studies are still needed to unravel the therapeutic potentials of compounds that selectively target SIRT6 in age-associated diseases including T2D [115].

Protein tyrosine phosphatases (PTPs) modulate signaling pathways by dephosphorylating phosphorylated tyrosine residues. PTP1B is a key negative regulator of insulin receptor and insulin receptor substrate 1 signaling intensity and duration [116]. In diabetics, PTP1B expression, is increased, and insulin signal is attenuated resulting from increased serine/threonine phosphorylation and decreased tyrosine phosphorylation of the insulin receptor [117]. Thus, PTP1B inhibitors are pursued as insulin sensitizing antidiabetic targets against type 2 diabetes mellitus and obesity. Clinically, reducing PTP1B expression enhances insulin signaling, improves glucose tolerance and reduces adipose tissue storage of triglyceride under conditions of over-nutrition. For these reasons, the pharmaceutical development of PTP1B inhibitors may serve as a novel type of insulin sensitizer in the management of type 2 diabetes and other cardiovascular syndrome or obesity [117]. Vitexin and isovitexin, the flavone derivatives of nothofagin, inhibited PTP1B activity with IC_50_ value of 7.62 ± 0.21 and 17.76 ± 0.53 μM, respectively, and apigenin with an IC_50_ value of 24.76 ± 2.17 μM [118]. A comparative study of aqueous and ethanolic green rooibos extracts showed potent PTP1B inhibitory activity with average IC_50_ values of 7.5 µg/mL and 7.7 µg/mL for the 80 and 60% ethanol-based extracts and 10 µg/mL for aqueous green rooibos extracts, respectively [119]. Principal component analysis of the flavonoid composition of the extracts and their PTP1B inhibitory activity indicated an association between inhibition of PTP1B and luteolin-7-*O*-glucoside, and this is supported by a study that demonstrated the inhibitory effect of luteolin on PTP1B with an IC_50_ value of 6.7 µM [120].

In addition to PTP1B, inhibition of megakaryocyte protein tyrosine phosphatase 2 (PTP-MEG2) enhances AMPK phosphorylation. Phloridzin inhibited PTP-MEG2 (IC_50_ = 32 ± 1.06 µM), enhanced AMPK and AKT activation and increased glucose uptake in 3T3-L1 adipocytes and in C2C12 skeletal muscle cells. Phloridzin also restored insulin signaling in palmitate-treated insulin resistant C2C12 and 3T3-L1 cells [121].

By modulating the physiological function of the kidney to promote glucosuria, SGLT2 inhibitors provide an alternative mechanism to control postprandial glucose. Suppressing glucose reabsorption by inhibiting SGLTs in the kidneys is an effective therapeutic strategy for treating type 2 diabetes. Selective inhibition of SGLT2 over SGLT1 is critical for minimizing adverse gastrointestinal side effects associated with SGLT1 inhibition [122]. Various modifications to phloridzin as lead compound for the development of synthetic analogs include a benzofuran ring moiety and aryl *C*-linked glycosides that alter the SLGT2 selectivity and increased selectivity towards SGLT2 [123,124]. Natural *C*-glucosyl dihydrochalcones such as nothofagin thus hold promise as they more potently inhibit SGLT2 than SGLT1 [125]. Molecular docking and dynamics simulations carried out on aspalathin, nothofagin, dapagliflozin (a synthetic *C*-aryl glucoside and analog of phloridzin) and SGLT2 complexes demonstrated similar results in terms of binding energies and binding modes for aspalathin and nothofagin to that of dapagliflozin [126].

Aspalathin was found to promote insulin secretion from cultured RIN-5F cells, a rat-derived pancreatic β-cell line. In these cells, aspalathin suppressed the increase in ROS, induced by advanced glycation end products (AGEs) and protected the cells from oxidative stress [108]. In a more recent study, the antioxidative effect of aspalathin was confirmed, with β-cell protection against glucotoxicity demonstrated as a result of increased expression of nuclear factor erythroid 2-related factor 2 (NRF2)-regulated antioxidant enzymes in the β-cell line, insulinoma 1E. Interestingly, aspalathin also suppressed the proapoptotic genes, Thioredoxin interacting protein (*Txnip*) and the DNA damage-inducible transcript 3 (*Ddit3*), and increased the expression of the protective antioxidant response gene, heme oxygenase 1 (*Hmox1*) [127]. TXNIP has recently emerged as a potential pancreatic β-cell-specific therapeutic target for T2D [128].

### 3.4. Targeting Gut Microbiota to Reduce Inflammation and Oxidative Stress

An underpinning factor in the anti-inflammatory and anti-oxidative properties of vitexin, phloretin and isoorientin is their capacity to modulate gut microbiota. The role of gut microbiota has come to the fore as a major role player in the development and progression of diabetes and other metabolic related diseases. Several reviews extensively discuss the significant role of microbial gut dysbiosis whereby host (human) intestinal barrier function is affected and disruption of metabolic homeostasis occurs; diet has been described as being an essential regulator of this process [129,130,131,132]. As much as a poor diet can result in damage to intestinal barrier function and dysregulation of gut microbiota, so too can nutritional factors such as natural flavonoids play a positive role. In fact, the commonly prescribed antidiabetic drug metformin, which was originally derived from *Galega officinalis*, has been shown to mediate its antidiabetic activity, in part, by increasing the abundance of probiotic and SCFA-producing microbiota species [133,134].

A recent study investigating the effect of isoorientin on the gut microbiota of BALB/c mice showed the compound to inhibit the growth of pathogenic gut microbes, including *Alistipes*, *Helicobacter*, and *Oscillibacter* [135]. These pathogenic species of gut microbiota are strongly associated with inflammation usually through the suppression of Th17 cells, which play a key role in antibody production and immune cell recruitment [136,137]. The BALB/c mice treated with isoorientin showed improved hepatic metabolism and oxidative parameters [135]. Isoorientin, at a concentration of 20 mg/kg BW, was also shown to reduce gut dysbiosis induced by a global food-borne pollutant (benzo[a]pyrene) by inducing changes in the abundance of *Faecalibaculum*, *Lactobacillus*, *Acinetobacter*, *Desulfovibrio* and, again, *Alistipes* [138].

Phloretin is renowned for its anti-bacterial and anti-inflammatory activities [139,140]. Again, this flavonoid was found to regulate, amongst others, *Alistipes* and *Oscillibacter* abundance in ulcerative colitis mice, with a concomitant improvement in colon inflammation, reduction in oxidative stress and maintenance of intestinal barrier integrity via the inhibition of NF-κB and NLRP3 inflammasome activation [141].

Vitexin has garnered interest in its anti-oxidative function and, specifically, protection against neuro-inflammation [130]. Although poorly absorbed in the gastro-intestinal tract, vitexin was shown to reduce the release of pro-inflammatory cytokines, TNF-α, interleukin-1β (IL-1β) and increase the release of the anti-inflammatory cytokine interleukin-10 (IL-10) in mice and immune cells stimulated with LPS [142,143]. The effect observed in mice has now been largely attributed to the effect of vitexin on gut microbiota modulation. In high-fat diet fed mice, vitexin (10 mg/kg BW) modulated five main microbial phyla, namely *Firmicutes*, *Verrucomicrobiota*, *Desulfobacterota*, *Bacteroideta* and *Actinobacteriota* [144]. In particular, the *Firmicutes* are prominent in obesity related disorders, and the *Verrucomicrobiota* species, *Akkermansia*, is closely related to glucose tolerance, intestinal barrier dysfunction and dyslipidemia [145,146]. The modulation of microbiota induced by vitexin in the high-fat diet fed mice correlated with a reduction in malondialdehyde content, increased antioxidant activity as manifested in increased activity and expression of superoxide dismutase and catalase. Furthermore, pro-inflammatory cytokine expression was reduced.

A distinct relationship exists between the regulation of gut microbiota and the reduction of both oxidative stress and inflammation, creating a niche area for natural flavonoids, such as vitexin, phloretin and isoorientin to exert further beneficial effects on glucose and lipid metabolism. In fact, an aspalathin-rich green rooibos extract was shown to increase the abundance of beneficial microbiota species (e.g., *Faecalibacterium prausnitzii, Eubacterium* spp., *Sutterella* spp., and *Dorea longicatena*) and suppress pathogenic species such as *Salmonella enterica* [147]. Interestingly, aspalathin on its own did not have as prominent an effect as the extract, suggesting a causative role for biotransformation of flavonoids by the gut microbiome, but also additive activity of other flavonoids.

### 3.5. Mitochondrial Dysfuction

Aspalathin, and its flavone derivatives, isoorientin, and orientin (10 µM) increased endogenous GSH and superoxide dismutase antioxidant levels and ameliorated the production of intracellular ROS resulting from antimycin A induced mitochondrial dysfunction in C2C12 myotubes. The expression of genes involved in mitochondrial function, such as uncoupling protein 2 (*Ucp 2*), mitochondrial complex 1/3, *Sirt1*, nuclear respiratory factor 1 (*Nrf 1*), and mitochondrial transcription factor (*Tfam*) suggests that aspalathin and its flavone derivatives could protect against mitochondrial dysfunction in C2C12 skeletal muscle cells [148]. Treatment of palmitate-induced insulin resistant HepG2/C3A liver cells with aspalathin (10 μM) effectively increased free fatty acid and glucose uptake facilitated by increased GLUT2 protein expression. Mechanistically, aspalathin enhanced the activation of insulin stimulated AKT, and increased AMPK, and carnitine palmitoyl transferase 1 (CPT1) protein. Seahorse assessment of mitochondrial bioenergetics showed that aspalathin treatment increased the oxygen consumption rate, thereby enhancing ATP production and basal, maximal and spare respiration capacity in these insulin-resistant cells [149]. Interestingly, a fermented rooibos extract with low levels of aspalathin (0.4%), and other antioxidants, induced similar ameliorative effects on impaired mitochondrial function in H9c2 cardiomyocytes under high glucose (33 mM) culture conditions. It was suggested that the rooibos extract (1 μg/mL for 6 h) enhanced mitochondrial energetics by increasing intracellular co-enzyme Q9 levels and reduced oxidative stress [150]. Although a direct correlation cannot be drawn between these experimental conditions, alternate mechanisms could account for these observed effects.

## 4. Conclusions

This review provided new insights into the potential antidiabetic activities of aspalathin and related compounds, focusing on recently published therapeutic targets, which include enhancing insulin signaling via AMPK and AKT activation and improving mitochondrial function. We also describe the capacity of these compounds to modulate insulin receptor and insulin receptor substrate 1 via PTP inhibition, to promote glucosuria via SGLT2 inhibition and to suppress hyperuricemia. Furthermore, selected compounds of interest suppress SIRT6 specifically, thereby improving insulin activation of AKT and increasing glycolysis and suppressing gluconeogenesis. The *C*-glycosyl parent compounds are active in cell models, but poor bioavailability would limit the therapeutic effects in vivo. Biotransformation in the gut is most likely responsible for enhancing therapeutic effects observed in animal models, and the gut microbiome has been shown to be an important mediator in the anti-inflammatory and antioxidative effects of these compounds. *C*-glycosyl compounds and their derivatives have the potential to regulate multiple factors associated with the development and progression of T2D; future research should focussed on compound derivatives and analogs with enhanced bioactivity.

## Figures and Tables

**Table 1:** Mean individual flavonoid content of infusions and extracts of fermented and green rooibos with the range in brackets.

Compound	Infusion (mg/L) ^1^		Extract (g/kg) ^2^	
	Fermented (*n* = 114) ^3^	Green (*n*= 29) ^4^	Fermented (*n*= 74) ^3^	Green (*n* = 10) ^4^
Aspalathin	5.8 (nd ^5^–15.7) ^6^	179 (76–255)	5.8 (1.6–15)	95 (54–116)
Nothofagin	1.0 (nd–2.8)	15 (7–25)	0.7 (0.3–1.8)	7.7 (3.6–12)
Orientin	11 (10–14)	15 (7.6–21)	7.9 (4.4–9.0)	8.6 (7.4–9.7)
Isoorientin	15 (7.4–21)	15 (7.6–21)	8.3 (4.7–10.3	9.1 (7.2–10.6)
Vitexin	2.3 (1.3–3.3)	2.4 (1.2–3.3)	nq ^5^	1.6 (1.2–1.8)
Isovitexin	2.4 (1.4–3.3)	3.0 (1.6–4.4)	nq	1.9 (1.5–2.2)

^1^ Prepared at ‘cup-of-tea’ strength (2.5 g/200 mL; infused in freshly boiled water for 5 min). ^2^ Hot water extract prepared at 1:10 solid: solvent (*m*/*v*) ratio with 30 min extraction at 93 °C. ^3^ Data for fermented rooibos from Joubert and De Beer [13]. ^4^ Data for green rooibos from Viraragavan et al. [15]. ^5^ ND—not detected; nq—not quantified. ^6^ Minimum to maximum values for a sample set.

**Table 2:** Bioavailability studies of aspalathin (ASP) and nothofagin (NOT) in human subjects after a single dose.

Dosage Form	ASP (mg) ^1^	NOT (mg) ^1^	Compound and Metabolites ^2^		ASP Excretion in Urine	Ref.
			**Plasma**	**Urine**		
Green rooibos infusion (300 mL; 14 g/L, added to boiling water and infused for 10 min)	91	nq ^3^	nd ^4^	Methylated ASP; methylated and glucuronidated ASP	Max. conc. reached <2 h after ingestion; 0.74% excreted during 0–24 h	[49]
Green rooibos ‘ready-to-drink’ beverage (500 mL)	41	7	nd	Glucuronidated ASP (2); methylated and glucuronidated ASP (3); methylated and sulphated ASP; sulphated ASP; NOT & metabolites not detected	Most excreted <5 h after ingestion; 0.22% excreted during 0–24 h	[50]
Fermented rooibos ‘ready-to-drink’ beverage (500 mL)	3.6	0.8	nd	methylated and glucuronidated ASP (3); methylated and sulphated ASP; sulphated ASP; NOT & metabolites not detected	0.09% excreted during 0–24 h	[50]
Green rooibos infusion (20 g/L; 10 min infused in freshly-boiled water)	287	34	ASP	ASP; glucuronidated ASP; methylated ASP; methylated and glucuronidated ASP (3); methylated and sulphated ASP; sulphated ASP; glucuronidated 3-hydroxyphoretin; NOT; glucuronidated NOT; glucuronidated phloretin	0.17% recovery rate at t_max_	[48]
Isolated fraction ^5^ of green rooibos, reconstituted in 500 mL water to similar phenolic content as green rooibos infusion			ASP	As for infusion, except for glucuronidated nothofagin	0.10% recovery rate at t_max_	[48]

^1^ Dose. ^2^ Number in brackets indicate number of metabolite derivatives. ^3^ Not quantified. ^4^ Not detected. ^5^ Fraction was isolated by high-speed counter-current chromatography; contained 0.3 g ASP/0.5 g fraction added to 500 mL water; fraction also contained nothofagin, orientin, isoorientin and isovitexin.

## Abbreviations

AKT	Protein kinase B
AMPK	5′-Adenosine monophosphate-activated protein kinase
ASP	Aspalathin
AUC_0-t_	Area under the curve
BCAAs	Branched-chain amino acids
BW	Body mass
C_max_	Maximum plasma concentration
DAG	Diacylglycerol
*Ddit3*	DNA damage-inducible transcript 3
DW	Dry weight
Fcr	Flavanone- and flavanonol-cleaving reductase
FOXO1	Forkhead box protein O1
GCN5	Non-repressed protein 5
GLUT2/4	Glucose transporter 2/4
*Hmox1*	Heme oxygenase 1
HOMA-IR	Homeostasis model assessment of insulin resistance
IL-1β	Interleukin 1 beta
IL-10	Interleukin 10
IMP	Inosine-5′-monophosphate
IPGTT	Intraperitoneal glucose tolerance test
IR	Insulin resistance
LPH	Lactase-phloridzin hydrolase
LPS	Lipopolysaccharide(s)
MRP2	Multidrug resistance protein 2
NFκB	Nuclear factor-κB
NLRP3	Nod-like receptor family pyrin domain containing 3
NOT	Nothofagin
*Nrf1*	Nuclear respiratory factor 1
NRF2	Nuclear factor erythroid 2-related factor 2
PKC	Protein kinase C
PDX1	Pancreatic and duodenal homeobox 1
PGC 1α	Peroxisome proliferator-activated receptor γ co-activator 1α
P-gp	P-glycoprotein
PTPs	Protein tyrosine phosphatases
PTP-MEG2	Megakaryocyte protein tyrosine phosphatase 2
ROS	Reactive oxygen species
SGLT1/2	Sodium-glucose co-transporter-1/2
*Sirt1*	Sirtuin 1
SIRT6	Sirtuin 6
SCFA	Short-chain fatty acid
t_½_	Elimination half-life
t_max_	Time at maximum absorption
*Tfam*	Mitochondrial transcription factor
TNF-α	Tumour necrosis factor-α
T2D	Type 2 diabetes
*Txnip*	Thioredoxin interacting protein
*Ucp2*	Uncoupling protein 2

## References

[1] International Diabetes Federation (2019). IDF Diabetes Atlas.

[2] Saeedi P., Petersohn I., Salpea P., Malanda B., Karuranga S., Unwin N., Colagiuri S., Guariguata L., Motala A.A., Ogurtsova K. (2019). IDF Diabetes Atlas Committee Global and Regional Diabetes Prevalence Estimates for 2019 and Projections for 2030 and 2045: Results from the International Diabetes Federation Diabetes Atlas, 9th Edition. Diabetes Res. Clin. Pract..

[3] Williams R., Karuranga S., Malanda B., Saeedi P., Basit A., Besançon S., Bommer C., Esteghamati A., Ogurtsova K., Zhang P. (2020). Global and Regional Estimates and Projections of Diabetes-Related Health Expenditure: Results from the International Diabetes Federation Diabetes Atlas, 9th Edition. Diabetes Res. Clin. Pract..

[4] Zhang M., Zhu S., Yang W., Huang Q., Ho C.-T. (2021). The Biological Fate and Bioefficacy of Citrus Flavonoids: Bioavailability, Biotransformation, and Delivery Systems. Food Funct..

[5] Stander M.A., Van Wyk B.-E., Taylor M.J.C., Long H.S. (2017). Analysis of Phenolic Compounds in Rooibos Tea (*Aspalathus linearis*) with a Comparison of Flavonoid-Based Compounds in Natural Populations of Plants from Different Regions. J. Agric. Food Chem..

[6] Mariadoss A.V.A., Vinyagam R., Rajamanickam V., Sankaran V., Venkatesan S., David E. (2019). Pharmacological Aspects and Potential Use of Phloretin: A Systemic Review. Mini Rev. Med. Chem..

[7] Tian L., Cao J., Zhao T., Liu Y., Khan A., Cheng G. (2021). The Bioavailability, Extraction, Biosynthesis and Distribution of Natural Dihydrochalcone: Phloridzin. Int. J. Mol. Sci..

[8] Muller C.J.F., Malherbe C.J., Chellan N., Yagasaki K., Miura Y., Joubert E. (2018). Potential of Rooibos, Its Major *C*-Glucosyl Flavonoids, and *Z*-2-(β-D-Glucopyranosyloxy)-3-Phenylpropenoic Acid in Prevention of Metabolic Syndrome. Crit. Rev. Food Sci. Nutr..

[9] Johnson R., de Beer D., Dludla P., Ferreira D., Muller C., Joubert E. (2018). Aspalathin from Rooibos (*Aspalathus linearis*): A Bioactive *C*-Glucosyl Dihydrochalcone with Potential to Target the Metabolic Syndrome. Planta Med..

[10] Dludla P.V., Joubert E., Muller C.J.F., Louw J., Johnson R. (2017). Hyperglycemia-Induced Oxidative Stress and Heart Disease-Cardioprotective Effects of Rooibos Flavonoids and Phenylpyruvic Acid-2-*O*-β-D-Glucoside. Nutr. Metab..

[11] Braune A., Blaut M. (2016). Bacterial Species Involved in the Conversion of Dietary Flavonoids in the Human Gut. Gut Microbes.

[12] Joubert E., de Beer D. (2011). Rooibos (*Aspalathus linearis*) beyond the Farm Gate: From Herbal Tea to Potential Phytopharmaceutical. S. Afr. J. Bot..

[13] Joubert E., de Beer D. (2014). Antioxidants of Rooibos Beverages. Processing and Impact on Antioxidants in Beverages.

[14] de Beer D., Miller N., Joubert E. (2017). Production of Dihydrochalcone-Rich Green Rooibos (*Aspalathus linearis*) Extract Taking into Account Seasonal and Batch-to-Batch Variation in Phenolic Composition of Plant Material. S. Afr. J. Bot..

[15] Viraragavan A., Hlengwa N., de Beer D., Riedel S., Miller N., Bowles S., Walczak B., Muller C., Joubert E. (2020). Model Development for Predicting in Vitro Bio-Capacity of Green Rooibos Extract Based on Composition for Application as Screening Tool in Quality Control. Food Funct..

[16] de Cassia Vilhena da Silva R., Bolda Mariano L.N., Bidinha E.R., Bueno de Almeida C.L., Cechinel-Filho V., Santos Zanuncio V.S., Silva D.B., Gasparotto Junior A., de Souza P. (2021). Ethyl Acetate Fraction from *Leandra dasytricha* (A. Gray) Cong. Leaves Promotes Vasodilatation and Reduces Blood Pressure in Normotensive and Hypertensive Rats. Evid.-Based Complement. Altern. Med..

[17] Rojas-Garbanzo C., Zimmermann B.F., Schulze-Kaysers N., Schieber A. (2017). Characterization of Phenolic and Other Polar Compounds in Peel and Flesh of Pink Guava (*Psidium guajava* L. Cv. ‘Criolla’) by Ultra-High Performance Liquid Chromatography with Diode Array and Mass Spectrometric Detection. Food Res. Int..

[18] Tan S., Wang Z., Xiang Y., Deng T., Zhao X., Shi S., Zheng Q., Gao X., Li W. (2020). The Effects of Drying Methods on Chemical Profiles and Antioxidant Activities of Two Cultivars of *Psidium guajava* Fruits. LWT—Food Sci. Technol..

[19] Niederberger K.E., Tennant D.R., Bellion P. (2020). Dietary Intake of Phloridzin from Natural Occurrence in Foods. Br. J. Nutr..

[20] Shang A., Liu H.-Y., Luo M., Xia Y., Yang X., Li H.-Y., Wu D.-T., Sun Q., Geng F., Li H.-B. (2020). Sweet Tea (*Lithocarpus polystachyus* Rehd.) as a New Natural Source of Bioactive Dihydrochalcones with Multiple Health Benefits. Crit. Rev. Food Sci. Nutr..

[21] Zielinska D., Szawara-Nowak D., Zielinski H. (2007). Comparison of Spectrophotometric and Electrochemical Methods for the Evaluation of the Antioxidant Capacity of Buckwheat Products after Hydrothermal Treatment. J. Agric. Food Chem..

[22] Li H., Cao D., Yi J., Cao J., Jiang W. (2012). Identification of the Flavonoids in Mungbean (*Phaseolus radiatus* L.) Soup and Their Antioxidant Activities. Food Chem..

[23] Paśko P., Sajewicz M., Gorinstein S., Zachwieja Z. (2008). Analysis of Selected Phenolic Acids and Flavonoids in *Amaranthus cruentus* and *Chenopodium* quinoa Seeds and Sprouts by HPLC. Acta Chromatogr..

[24] He X., Luan F., Yang Y., Wang Z., Zhao Z., Fang J., Wang M., Zuo M., Li Y. (2020). *Passiflora edulis*: An Insight into Current Researches on Phytochemistry and Pharmacology. Front. Pharmacol..

[25] da Silveira T.F.F., de Souza T.C.L., Carvalho A.V., Ribeiro A.B., Kuhnle G.G.C., Godoy H.T. (2017). White Açaí Juice (*Euterpe oleracea*): Phenolic Composition by LC-ESI-MS/MS, Antioxidant Capacity and Inhibition Effect on the Formation of Colorectal Cancer Related Compounds. J. Funct. Foods.

[26] Zhang W., Zhu Y., Liu Q., Bao J., Liu Q. (2017). Identification and Quantification of Polyphenols in Hull, Bran and Endosperm of Common Buckwheat (*Fagopyrum esculentum*) Seeds. J. Funct. Foods.

[27] Zhang X., Shang P., Qin F., Zhou Q., Gao B., Huang H., Yang H., Shi H., (Lucy) Yu L. (2013). Chemical Composition and Antioxidative and Anti-Inflammatory Properties of Ten Commercial Mung Bean Samples. LWT—Food Sci. Technol..

[28] Zeraik M.L., Yariwake J.H. (2010). Quantification of Isoorientin and Total Flavonoids in *Passiflora edulis* Fruit Pulp by HPLC-UV/DAD. Microchem. J..

[29] Sanchez B.A.O., Celestino S.M.C., de Abreu Gloria M.B., Celestino I.C., Lozada M.I.O., Júnior S.D.A., de Alencar E.R., de Lacerda de Oliveira L. (2020). Pasteurization of Passion Fruit *Passiflora setacea* Pulp to Optimize Bioactive Compounds Retention. Food Chem. X.

[30] Human C., Danton O., de Beer D., Maruyama T., Alexander L., Malherbe C., Hamburger M., Joubert E. (2021). Identification of a Novel Di-*C*-Glycosyl Dihydrochalcone and the Thermal Stability of Polyphenols in Model Ready-to-Drink Beverage Solutions with *Cyclopia subternata* Extract as Functional Ingredient. Food Chem..

[31] Sadilova E., Stintzing F.C., Kammerer D.R., Carle R. (2009). Matrix Dependent Impact of Sugar and Ascorbic Acid Addition on Color and Anthocyanin Stability of Black Carrot, Elderberry and Strawberry Single Strength and from Concentrate Juices upon Thermal Treatment. Food Res. Int..

[32] Capuano E., Oliviero T., van Boekel M.A.J.S. (2018). Modeling Food Matrix Effects on Chemical Reactivity: Challenges and Perspectives. Crit. Rev. Food Sci. Nutr..

[33] Walters N.A., de Villiers A., Joubert E., de Beer D. (2017). Improved HPLC Method for Rooibos Phenolics Targeting Changes Due to Fermentation. J. Food Compos. Anal..

[34] de Beer D., Tobin J., Walczak B., van der Rijst M., Joubert E. (2019). Phenolic Composition of Rooibos Changes during Simulated Fermentation: Effect of Endogenous Enzymes and Fermentation Temperature on Reaction Kinetics. Food Res. Int..

[35] Miller N., de Beer D., Aucamp M., Malherbe C.J., Joubert E. (2018). Inulin as Microencapsulating Agent Improves Physicochemical Properties of Spray-Dried Aspalathin-Rich Green Rooibos (*Aspalathus linearis*) Extract with α-Glucosidase Inhibitory Activity. J. Funct. Foods.

[36] Human C., de Beer D., Muller M., van der Rijst M., Aucamp M., Tredoux A., de Villiers A., Joubert E. (2021). Shelf-Life Stability of Ready-to-Use Green Rooibos Iced Tea Powder—Assessment of Physical, Chemical, and Sensory Properties. Molecules.

[37] Pacheco-Palencia L.A., Duncan C.E., Talcott S.T. (2009). Phytochemical Composition and Thermal Stability of Two Commercial Açai Species, *Euterpe oleracea* and *Euterpe precatoria*. Food Chem..

[38] de Paepe D., Valkenborg D., Coudijzer K., Noten B., Servaes K., de Loose M., Voorspoels S., Diels L., van Droogenbroeck B. (2014). Thermal Degradation of Cloudy Apple Juice Phenolic Constituents. Food Chem..

[39] van der Sluis A.A., Dekker M., van Boekel M.A.J.S. (2005). Activity and Concentration of Polyphenolic Antioxidants in Apple Juice. 3. Stability during Storage. J. Agric. Food Chem..

[40] Heras-Ramírez M.E., Quintero-Ramos A., Camacho-Dávila A.A., Barnard J., Talamás-Abbud R., Torres-Muñoz J.V., Salas-Muñoz E. (2012). Effect of Blanching and Drying Temperature on Polyphenolic Compound Stability and Antioxidant Capacity of Apple Pomace. Food Bioprocess Technol..

[41] Lavelli V., Vantaggi C. (2009). Rate of Antioxidant Degradation and Color Variations in Dehydrated Apples as Related to Water Activity. J. Agric. Food Chem..

[42] Lavelli V., Corti S. (2011). Phloridzin and Other Phytochemicals in Apple Pomace: Stability Evaluation upon Dehydration and Storage of Dried Product. Food Chem..

[43] Gulsunoglu Z., Karbancioglu-Guler F., Raes K., Kilic-Akyilmaz M. (2019). Soluble and Insoluble-Bound Phenolics and Antioxidant Activity of Various Industrial Plant Wastes. Int. J. Food Prop..

[44] Sęczyk Ł., Gawlik-Dziki U., Świeca M. (2021). Influence of Phenolic-Food Matrix Interactions on In Vitro Bioaccessibility of Selected Phenolic Compounds and Nutrients Digestibility in Fortified White Bean Paste. Antioxidants.

[45] Zhang B., Zhang Y., Li H., Deng Z., Tsao R. (2020). A Review on Insoluble-Bound Phenolics in Plant-Based Food Matrix and Their Contribution to Human Health with Future Perspectives. Trends Food Sci. Technol..

[46] Owens J. (2003). Chris Lipinski Discusses Life and Chemistry after the Rule of Five. Drug Discov. Today.

[47] Courts F.L., Williamson G. (2015). The Occurrence, Fate and Biological Activities of *C*-Glycosyl Flavonoids in the Human Diet. Crit. Rev. Food Sci. Nutr..

[48] Breiter T., Laue C., Kressel G., Gröll S., Engelhardt U.H., Hahn A. (2011). Bioavailability and Antioxidant Potential of Rooibos Flavonoids in Humans Following the Consumption of Different Rooibos Formulations. Food Chem..

[49] Courts F.L., Williamson G. (2009). The *C*-Glycosyl Flavonoid, Aspalathin, Is Absorbed, Methylated and Glucuronidated Intact in Humans. Mol. Nutr. Food Res..

[50] Stalmach A., Mullen W., Pecorari M., Serafini M., Crozier A. (2009). Bioavailability of *C*-Linked Dihydrochalcone and Flavanone Glucosides in Humans Following Ingestion of Unfermented and Fermented Rooibos Teas. J. Agric. Food Chem..

[51] Kreuz S., Joubert E., Waldmann K.-H., Ternes W. (2008). Aspalathin, a Flavonoid in *Aspalathus linearis* (Rooibos), Is Absorbed by Pig Intestine as a *C*-Glycoside. Nutr. Res..

[52] Bowles S., Joubert E., de Beer D., Louw J., Brunschwig C., Njoroge M., Lawrence N., Wiesner L., Chibale K., Muller C. (2017). Intestinal Transport Characteristics and Metabolism of *C*-Glucosyl Dihydrochalcone, Aspalathin. Molecules.

[53] Damiani E., Carloni P., Rocchetti G., Senizza B., Tiano L., Joubert E., de Beer D., Lucini L. (2019). Impact of Cold versus Hot Brewing on the Phenolic Profile and Antioxidant Capacity of Rooibos (*Aspalathus linearis*) Herbal Tea. Antioxidants.

[54] Zhang Y., Tie X., Bao B., Wu X., Zhang Y. (2007). Metabolism of Flavone *C*-Glucosides and *p*-Coumaric Acid from Antioxidant of Bamboo Leaves (AOB) in Rats. Br. J. Nutr..

[55] Li X., Huo T., Qin F., Lu X., Li F. (2007). Determination and Pharmacokinetics of Orientin in Rabbit Plasma by Liquid Chromatography after Intravenous Administration of Orientin and *Trollius chinensis* Bunge Extract. J. Chromatogr. B.

[56] Li D., Wang Q., Yuan Z., Zhang L., Xu L., Cui Y., Duan K. (2008). Pharmacokinetics and Tissue Distribution Study of Orientin in Rat by Liquid Chromatography. J. Pharm. Biomed. Anal..

[57] Li D., Wang Q., Xu L., Li M., Jing X., Zhang L. (2008). Pharmacokinetic Study of Three Active Flavonoid Glycosides in Rat after Intravenous Administration of *Trollius ledebourii* Extract by Liquid Chromatography. Biomed. Chromatogr..

[58] Sun Y., Tsao R., Chen F., Li H., Wang J., Peng H., Zhang K., Deng Z. (2017). The Phytochemical Composition, Metabolites, Bioavailability and in Vivo Antioxidant Activity of *Tetrastigma hemsleyanum* Leaves in Rats. J. Funct. Foods.

[59] Tremmel M., Kiermaier J., Heilmann J. (2021). In Vitro Metabolism of Six *C*-Glycosidic Flavonoids from *Passiflora incarnata* L.. Int. J. Mol. Sci..

[60] Németh K., Plumb G.W., Berrin J.-G., Juge N., Jacob R., Naim H.Y., Williamson G., Swallow D.M., Kroon P.A. (2003). Deglycosylation by Small Intestinal Epithelial Cell α-Glucosidases Is a Critical Step in the Absorption and Metabolism of Dietary Flavonoid Glycosides in Humans. Eur. J. Nutr..

[61] Wang L., Li X., Mi L., Shen X., Feng T., Liu X., Wang Q. (2019). Study on Pharmacokinetics, Tissue Distribution, and Excretion of Phloretin and Its Prodrug 2′,4′,6′,4-Tetra-*O*-Acetylphloretin in Rats Using LC–MS/MS. Acta Chromatogr..

[62] Zhao Y.Y., Fan Y., Wang M., Wang J., Cheng J.X., Zou J.B., Zhang X.F., Shi Y.J., Guo D.Y. (2020). Studies on Pharmacokinetic Properties and Absorption Mechanism of Phloretin: In Vivo and in Vitro. Biomed. Pharmacother..

[63] Crespy V., Aprikian O., Morand C., Besson C., Manach C., Demigné C., Rémésy C. (2001). Bioavailability of Phloretin and Phloridzin in Rats. J. Nutr..

[64] Wang Z., Gao Z., Wang A., Jia L., Zhang X., Fang M., Yi K., Li Q., Hu H. (2019). Comparative Oral and Intravenous Pharmacokinetics of Phlorizin in Rats Having Type 2 Diabetes and in Normal Rats Based on Phase II Metabolism. Food Funct..

[65] Stracke B.A., Rüfer C.E., Bub A., Seifert S., Weibel F.P., Kunz C., Watzl B. (2010). No Effect of the Farming System (Organic/Conventional) on the Bioavailability of Apple (*Malus domestica* Bork., Cultivar Golden Delicious) Polyphenols in Healthy Men: A Comparative Study. Eur. J. Nutr..

[66] Mullen W., Borges G., Lean M.E.J., Roberts S.A., Crozier A. (2010). Identification of Metabolites in Human Plasma and Urine after Consumption of a Polyphenol-Rich Juice Drink. J. Agric. Food Chem..

[67] Rodríguez-Daza M.C., Pulido-Mateos E.C., Lupien-Meilleur J., Guyonnet D., Desjardins Y., Roy D. (2021). Polyphenol-Mediated Gut Microbiota Modulation: Toward Prebiotics and Further. Front. Nutr..

[68] Braune A., Gütschow M., Blaut M. (2019). An NADH-Dependent Reductase from *Eubacterium ramulus* Catalyzes the Stereospecific Heteroring Cleavage of Flavanones and Flavanonols. Appl. Environ. Microbiol..

[69] Maurer J.M., Schellekens R.C.A., van Rieke H.M., Wanke C., Iordanov V., Stellaard F., Wutzke K.D., Dijkstra G., van der Zee M., Woerdenbag H.J. (2015). Gastrointestinal PH and Transit Time Profiling in Healthy Volunteers Using the IntelliCap System Confirms Ileo-Colonic Release of ColoPulse Tablets. PLoS ONE.

[70] Wei B., Wang Y.-K., Qiu W.-H., Wang S.-J., Wu Y.-H., Xu X.-W., Wang H. (2020). Discovery and Mechanism of Intestinal Bacteria in Enzymatic Cleavage of C–C Glycosidic Bonds. Appl. Microbiol. Biotechnol..

[71] Braune A., Blaut M. (2012). Intestinal Bacterium *Eubacterium cellulosolvens*. Deglycosylates Flavonoid *C*- and *O*-Glucosides. Appl. Environ. Microbiol..

[72] Braune A., Blaut M. (2011). Deglycosylation of Puerarin and Other Aromatic *C*-Glucosides by a Newly Isolated Human Intestinal Bacterium: A Newly Isolated *C*-Glucoside-Cleaving Bacterium. Environ. Microbiol..

[73] Zheng S., Geng D., Liu S., Wang Q., Liu S., Wang R. (2019). A Newly Isolated Human Intestinal Bacterium Strain Capable of Deglycosylating Flavone *C*-Glycosides and Its Functional Properties. Microb. Cell Fact..

[74] Goris T., Cuadrat R.R.C., Braune A. (2021). Flavonoid-Modifying Capabilities of the Human Gut Microbiome—An In Silico Study. Nutrients.

[75] Herles C., Braune A., Blaut M. (2004). First Bacterial Chalcone Isomerase Isolated from *Eubacterium ramulus*. Arch. Microbiol..

[76] Gall M., Thomsen M., Peters C., Pavlidis I., Jonczyk P., Grünert P., Beutel S., Scheper T., Gross E., Backes M. (2014). Enzymatic Conversion of Flavonoids Using Bacterial Chalcone Isomerase and Enoate Reductase. Angew. Chem. Int. Ed. Engl..

[77] Schoefer L., Braune A., Blaut M. (2004). Cloning and Expression of a Phloretin Hydrolase Gene from *Eubacterium ramulus* and Characterization of the Recombinant Enzyme. Appl. Environ. Microbiol..

[78] Braune A., Engst W., Blaut M. (2005). Degradation of Neohesperidin Dihydrochalcone by Human Intestinal Bacteria. J. Agric. Food Chem..

[79] Yang G., Hong S., Yang P., Sun Y., Wang Y., Zhang P., Jiang W., Gu Y. (2021). Discovery of an Ene-Reductase for Initiating Flavone and Flavonol Catabolism in Gut Bacteria. Nat. Commun..

[80] Braune A., Gütschow M., Engst W., Blaut M. (2001). Degradation of Quercetin and Luteolin by *Eubacterium ramulus*. Appl. Environ. Microbiol..

[81] Schoefer L., Mohan R., Schwiertz A., Braune A., Blaut M. (2003). Anaerobic Degradation of Flavonoids by *Clostridium orbiscindens*. Appl. Environ. Microbiol..

[82] Galicia-Garcia U., Benito-Vicente A., Jebari S., Larrea-Sebal A., Siddiqi H., Uribe K.B., Ostolaza H., Martín C. (2020). Pathophysiology of Type 2 Diabetes Mellitus. Int. J. Mol. Sci..

[83] Sergi D., Naumovski N., Heilbronn L.K., Abeywardena M., O’Callaghan N., Lionetti L., Luscombe-Marsh N. (2019). Mitochondrial (Dys)Function and Insulin Resistance: From Pathophysiological Molecular Mechanisms to the Impact of Diet. Front. Physiol..

[84] Bruns I., Sauer B., Burger M.C., Eriksson J., Hofmann U., Braun Y., Harter P.N., Luger A.-L., Ronellenfitsch M.W., Steinbach J.P. (2019). Disruption of Peroxisome Proliferator–Activated Receptor γ Coactivator (PGC)-1α Reverts Key Features of the Neoplastic Phenotype of Glioma Cells. J. Biol. Chem..

[85] Ormazabal V., Nair S., Elfeky O., Aguayo C., Salomon C., Zuñiga F.A. (2018). Association between Insulin Resistance and the Development of Cardiovascular Disease. Cardiovasc. Diabetol..

[86] Pina A.F., Borges D.O., Meneses M.J., Branco P., Birne R., Vilasi A., Macedo M.P. (2020). Insulin: Trigger and Target of Renal Functions. Front. Cell Developm. Biol..

[87] Gill A., Kukreja S., Malhotra N., Chhabra N. (2013). Correlation of the Serum Insulin and the Serum Uric Acid Levels with the Glycated Haemoglobin Levels in the Patients of Type 2 Diabetes Mellitus. J. Clin. Diagn. Res..

[88] Bardin T., Richette P. (2014). Definition of Hyperuricemia and Gouty Conditions. Curr. Opin. Rheumatol..

[89] Zhu Y., Hu Y., Huang T., Zhang Y., Li Z., Luo C., Luo Y., Yuan H., Hisatome I., Yamamoto T. (2014). High Uric Acid Directly Inhibits Insulin Signalling and Induces Insulin Resistance. Biochem. Biophys. Res. Commun..

[90] Bahadoran Z., Mirmiran P., Kashfi K., Ghasemi A. (2021). Hyperuricemia-Induced Endothelial Insulin Resistance: The Nitric Oxide Connection. Pflugers Arch..

[91] Kondo M., Hirano Y., Nishio M., Furuya Y., Nakamura H., Watanabe T. (2013). Xanthine Oxidase Inhibitory Activity and Hypouricemic Effect of Aspalathin from Unfermented Rooibos. J. Food Sci..

[92] Adachi S.-I., Yoshizawa F., Yagasaki K. (2017). Hyperuricemia in Type 2 Diabetic Model KK-A^y^/Ta Mice: A Potent Animal Model with Positive Correlation between Insulin Resistance and Plasma High Uric Acid Levels. BMC Res. Notes.

[93] Kondo S., Adachi S., Yoshizawa F., Yagasaki K. (2021). Antidiabetic Effect of Taxifolin in Cultured L6 Myotubes and Type 2 Diabetic Model KK-A^y^/Ta Mice with Hyperglycemia and Hyperuricemia. Curr. Issues Mol. Biol..

[94] Adachi S., Nihei K., Ishihara Y., Yoshizawa F., Yagasaki K. (2017). Anti-Hyperuricemic Effect of Taxifolin in Cultured Hepatocytes and Model Mice. Cytotechnology.

[95] Martínez-Sánchez F.D., Vargas-Abonce V.P., Guerrero-Castillo A.P., Santos-Villavicencio M.D.L., Eseiza-Acevedo J., Meza-Arana C.E., Gulias-Herrero A., Gómez-Sámano M.Á. (2021). Serum Uric Acid Concentration is Associated with Insulin Resistance and Impaired Insulin Secretion in Adults at Risk for Type 2 Diabetes. Prim. Care Diabetes.

[96] Dhanya R., Arya A.D., Nisha P., Jayamurthy P. (2017). Quercetin, a Lead Compound against Type 2 Diabetes Ameliorates Glucose Uptake via AMPK Pathway in Skeletal Muscle Cell Line. Front. Pharmacol..

[97] Jiang H., Yamashita Y., Nakamura A., Croft K., Ashida H. (2019). Quercetin and Its Metabolite Isorhamnetin Promote Glucose Uptake through Different Signalling Pathways in Myotubes. Sci. Rep..

[98] Shi Y., Williamson G. (2016). Quercetin Lowers Plasma Uric Acid in Pre-Hyperuricaemic Males: A Randomised, Double-Blinded, Placebo-Controlled, Cross-over Trial. Br. J. Nutr..

[99] Adachi S.-I., Kondo S., Sato Y., Yoshizawa F., Yagasaki K. (2019). Anti-Hyperuricemic Effect of Isorhamnetin in Cultured Hepatocytes and Model Mice: Structure-Activity Relationships of Methylquercetins as Inhibitors of Uric Acid Production. Cytotechnology.

[100] Komatsu W., Kishi H., Yagasaki K., Ohhira S. (2018). Urolithin A Attenuates Pro-Inflammatory Mediator Production by Suppressing PI3-K/Akt/NF-ΚB and JNK/AP-1 Signaling Pathways in Lipopolysaccharide-Stimulated RAW264 Macrophages: Possible Involvement of NADPH Oxidase-Derived Reactive Oxygen Species. Eur. J. Pharmacol..

[101] Adachi S., Sasaki K., Kondo S., Komatsu W., Yoshizawa F., Isoda H., Yagasaki K. (2020). Antihyperuricemic Effect of Urolithin A in Cultured Hepatocytes and Model Mice. Molecules.

[102] Adachi S.-I., Oyama M., Kondo S., Yagasaki K. (2021). Comparative Effects of Quercetin, Luteolin, Apigenin and Their Related Polyphenols on Uric Acid Production in Cultured Hepatocytes and Suppression of Purine Bodies-Induced Hyperuricemia by Rutin in Mice. Cytotechnology.

[103] Yanai H., Adachi H., Hakoshima M., Katsuyama H. (2021). Molecular Biological and Clinical Understanding of the Pathophysiology and Treatments of Hyperuricemia and Its Association with Metabolic Syndrome, Cardiovascular Diseases and Chronic Kidney Disease. Int. J. Mol. Sci..

[104] Ghasemi A. (2021). Uric Acid-Induced Pancreatic β-Cell Dysfunction. BMC Endocr. Disord..

[105] Saltiel A.R., Kahn C.R. (2001). Insulin Signalling and the Regulation of Glucose and Lipid Metabolism. Nature.

[106] Qu Z., Zhou S., Li P., Liu C., Yuan B., Zhang S., Liu A. (2021). Natural Products and Skeletal Muscle Health. J. Nutr. Biochem..

[107] Kawano A., Nakamura H., Hata S., Minakawa M., Miura Y., Yagasaki K. (2009). Hypoglycemic Effect of Aspalathin, a Rooibos Tea Component from *Aspalathus linearis*, in Type 2 Diabetic Model db/db Mice. Phytomedicine.

[108] Son M.J., Minakawa M., Miura Y., Yagasaki K. (2013). Aspalathin Improves Hyperglycemia and Glucose Intolerance in Obese Diabetic ob/ob Mice. Eur. J. Nutr..

[109] Yagasaki K., Walrand S. (2019). Phytochemicals, Their Intestinal Metabolites, and Skeletal Muscle Function. Nutrition and Skeletal Muscle.

[110] Kamakura R., Son M.J., de Beer D., Joubert E., Miura Y., Yagasaki K. (2015). Antidiabetic Effect of Green Rooibos (*Aspalathus linearis*) Extract in Cultured Cells and Type 2 Diabetic Model KK-A^y^ Mice. Cytotechnology.

[111] Sociali G., Magnone M., Ravera S., Damonte P., Vigliarolo T., Von Holtey M., Vellone V.G., Millo E., Caffa I., Cea M. (2017). Pharmacological Sirt6 Inhibition Improves Glucose Tolerance in a Type 2 Diabetes Mouse Model. FASEB J..

[112] Mutlu B., Puigserver P. (2021). GCN5 Acetyltransferase in Cellular Energetic and Metabolic Processes. BBA-Gene Regul. Mech..

[113] Kuang J., Chen L., Tang Q., Zhang J., Li Y., He J. (2018). The Role of SIRT6 in Obesity and Diabetes. Front. Physiol..

[114] Rahnasto-Rilla M., Kokkola T., Jarho E., Lahtela-Kakkonen M., Moaddel R. (2016). N-Acylethanolamines Bind to SIRT6. ChemBioChem.

[115] Heger V., Tyni J., Hunyadi A., Horáková L., Lahtela-Kakkonen M., Rahnasto-Rilla M. (2019). Quercetin Based Derivatives as Sirtuin Inhibitors. Biomed. Pharmacother..

[116] Proença C., Freitas M., Ribeiro D., Sousa J.L.C., Carvalho F., Silva A.M.S., Fernandes P.A., Fernandes E. (2018). Inhibition of Protein Tyrosine Phosphatase 1B by Flavonoids: A Structure—Activity Relationship Study. Food Chem. Toxicol..

[117] Tiwari N. (2014). Therapeutic Targets for Diabetes Mellitus: An Update. Clin. Pharmacol. Biopharm..

[118] Choi J.S., Islam M.N., Ali M.Y., Kim E.J., Kim Y.M., Jung H.A. (2014). Effects of *C*-Glycosylation on Anti-Diabetic, Anti-Alzheimer’s Disease and Anti-Inflammatory Potential of Apigenin. Food Chem. Toxicol..

[119] Viraragavan A. (2017). Assessment of Chemical Markers as Surrogates for Efficacy and Safety of Rooibos Extracts. Master’s Thesis.

[120] Choi J.S., Islam M.N., Ali M.Y., Kim Y.M., Park H.J., Sohn H.S., Jung H.A. (2014). The Effects of *C*-Glycosylation of Luteolin on Its Antioxidant, Anti-Alzheimer’s Disease, Anti-Diabetic, and Anti-Inflammatory Activities. Arch. Pharm. Res..

[121] Yoon S.-Y., Yu J.S., Hwang J.Y., So H.M., Seo S.O., Kim J.K., Jang T.S., Chung S.J., Kim K.H. (2021). Phloridzin Acts as an Inhibitor of Protein-Tyrosine Phosphatase MEG2 Relevant to Insulin Resistance. Molecules.

[122] Dominguez Rieg J.A., Rieg T. (2019). What Does Sodium-Glucose Co-Transporter 1 Inhibition Add: Prospects for Dual Inhibition. Diabetes Obes. Metab..

[123] Dudash J., Zhang X., Zeck R.E., Johnson S.G., Cox G.G., Conway B.R., Rybczynski P.J., Demarest K.T. (2004). Glycosylated Dihydrochalcones as Potent and Selective Sodium Glucose Co-Transporter 2 (SGLT2) Inhibitors. Bioorg. Med. Chem. Lett..

[124] Ho L.-T., Kulkarni S.S., Lee J.-C. (2011). Development of Sodium-Dependent Glucose Co-Transporter 2 Inhibitors as Potential Anti-Diabetic Therapeutics. Curr. Top. Med. Chem..

[125] Jesus A.R., Vila-Viçosa D., Machuqueiro M., Marques A.P., Dore T.M., Rauter A.P. (2017). Targeting Type 2 Diabetes with *C*-Glucosyl Dihydrochalcones as Selective Sodium Glucose Co-Transporter 2 (SGLT2) Inhibitors: Synthesis and Biological Evaluation. J. Med. Chem..

[126] Liu W., Wang H., Meng F. (2015). In Silico Modeling of Aspalathin and Nothofagin against SGLT2. J. Theor. Comput. Chem..

[127] Moens C., Bensellam M., Himpe E., Muller C.J.F., Jonas J., Bouwens L. (2020). Aspalathin Protects Insulin-Producing β Cells against Glucotoxicity and Oxidative Stress-Induced Cell Death. Mol. Nutr. Food Res..

[128] Wondafrash D.Z., Nire’a A.T., Tafere G.G., Desta D.M., Berhe D.A., Zewdie K.A. (2020). Thioredoxin-Interacting Protein as a Novel Potential Therapeutic Target in Diabetes Mellitus and Its Underlying Complications. Diabetes Metab. Syndr. Obes. Targets Ther..

[129] Hou K., Zhang S., Wu Z., Zhu D., Chen F., Lei Z.-N., Liu W., Xiao C., Chen Z.-S. (2021). Reconstruction of Intestinal Microecology of Type 2 Diabetes by Fecal Microbiota Transplantation: Why and How. Bosn. J. Basic Med. Sci..

[130] Li W.-Z., Stirling K., Yang J.-J., Zhang L. (2020). Gut Microbiota and Diabetes: From Correlation to Causality and Mechanism. World J. Diabetes.

[131] Sharma S., Tripathi P. (2019). Gut Microbiome and Type 2 Diabetes: Where We Are and Where to Go?. J. Nutr. Biochem..

[132] Xi Y., Xu P.-F. (2021). Diabetes and Gut Microbiota. World J. Diabetes.

[133] Guo G.L., Xie W. (2018). Metformin Action through the Microbiome and Bile Acids. Nat. Med..

[134] Wu H., Esteve E., Tremaroli V., Khan M.T., Caesar R., Mannerås-Holm L., Ståhlman M., Olsson L.M., Serino M., Planas-Fèlix M. (2017). Metformin Alters the Gut Microbiome of Individuals with Treatment-Naive Type 2 Diabetes, Contributing to the Therapeutic Effects of the Drug. Nat. Med..

[135] Yuan L., Li X., He S., Gao C., Wang C., Shao Y. (2018). Effects of Natural Flavonoid Isoorientin on Growth Performance and Gut Microbiota of Mice. J. Agric. Food Chem..

[136] Jiang W., Wu N., Wang X., Chi Y., Zhang Y., Qiu X., Hu Y., Li J., Liu Y. (2015). Dysbiosis Gut Microbiota Associated with Inflammation and Impaired Mucosal Immune Function in Intestine of Humans with Non-Alcoholic Fatty Liver Disease. Sci. Rep..

[137] Tesmer L.A., Lundy S.K., Sarkar S., Fox D.A. (2008). Th17 Cells in Human Disease. Immunol. Rev..

[138] He S., Li X., Li C., Deng H., Shao Y., Yuan L. (2019). Isoorientin Attenuates Benzo[a]Pyrene-Induced Colonic Injury and Gut Microbiota Disorders in Mice. Food Res. Int..

[139] Barreca D., Bellocco E., Laganà G., Ginestra G., Bisignano C. (2014). Biochemical and Antimicrobial Activity of Phloretin and Its Glycosilated Derivatives Present in Apple and Kumquat. Food Chem..

[140] Chang W.-T., Huang W.-C., Liou C.-J. (2012). Evaluation of the Anti-Inflammatory Effects of Phloretin and Phlorizin in Lipopolysaccharide-Stimulated Mouse Macrophages. Food Chem..

[141] Wu M., Li P., An Y., Ren J., Yan D., Cui J., Li D., Li M., Wang M., Zhong G. (2019). Phloretin Ameliorates Dextran Sulfate Sodium-Induced Ulcerative Colitis in Mice by Regulating the Gut Microbiota. Pharmacol. Res..

[142] Ninfali P., Dominici S., Angelino D., Gennari L., Buondelmonte C., Giorgi L. (2013). An Enzyme-Linked Immunosorbent Assay for the Measurement of Plasma Flavonoids in Mice Fed Apigenin-*C*-Glycoside. J. Sci. Food Agric..

[143] Rosa S.I.G., Rios-Santos F., Balogun S.O., de Oliveira Martins D.T. (2016). Vitexin Reduces Neutrophil Migration to Inflammatory Focus by Down-Regulating pro-Inflammatory Mediators via Inhibition of P38, ERK1/2 and JNK Pathway. Phytomedicine.

[144] Li S., Liang T., Zhang Y., Huang K., Yang S., Lv H., Chen Y., Zhang C., Guan X. (2021). Vitexin Alleviates High-Fat Diet Induced Brain Oxidative Stress and Inflammation via Anti-Oxidant, Anti-Inflammatory and Gut Microbiota Modulating Properties. Free Radic. Biol. Med..

[145] Ottman N., Geerlings S.Y., Aalvink S., de Vos W.M., Belzer C. (2017). Action and Function of *Akkermansia muciniphila* in Microbiome Ecology, Health and Disease. Best Pract. Res. Clin. Gastroenterol..

[146] Turnbaugh P.J., Ley R.E., Mahowald M.A., Magrini V., Mardis E.R., Gordon J.I. (2006). An Obesity-Associated Gut Microbiome with Increased Capacity for Energy Harvest. Nature.

[147] Mangwana N. (2020). The in Vitro Faecal Evaluation of Prebiotic Effects of Rooibos Phenolic Compounds on the Gut Microbiota of Vervet Monkeys (*Chlorocebus Pygerythrus*). Master’s Thesis.

[148] Mthembu S.X.H., Muller C.J.F., Dludla P.V., Madoroba E., Kappo A.P., Mazibuko-Mbeje S.E. (2021). Rooibos Flavonoids, Aspalathin, Isoorientin, and Orientin Ameliorate Antimycin A-Induced Mitochondrial Dysfunction by Improving Mitochondrial Bioenergetics in Cultured Skeletal Muscle Cells. Molecules.

[149] Mazibuko-Mbeje S.E., Dludla P.V., Johnson R., Joubert E., Louw J., Ziqubu K., Tiano L., Silvestri S., Orlando P., Opoku A.R. (2019). Aspalathin, a Natural Product with the Potential to Reverse Hepatic Insulin Resistance by Improving Energy Metabolism and Mitochondrial Respiration. PLoS ONE.

[150] Dludla P.V., Johnson R., Mazibuko-Mbeje S.E., Muller C.J.F., Louw J., Joubert E., Orlando P., Silvestri S., Chellan N., Nkambule B.B. (2020). Fermented Rooibos Extract Attenuates Hyperglycemia-Induced Myocardial Oxidative Damage by Improving Mitochondrial Energetics and Intracellular Antioxidant Capacity. S. Afr. J. Bot..

